# A Pathway Proteomic Profile of Ischemic Stroke Survivors Reveals Innate Immune Dysfunction in Association with Mild Symptoms of Depression – A Pilot Study

**DOI:** 10.3389/fneur.2016.00085

**Published:** 2016-06-14

**Authors:** Vinh A. Nguyen, Leeanne M. Carey, Loretta Giummarra, Pierre Faou, Ira Cooke, David W. Howells, Tamara Tse, S. Lance Macaulay, Henry Ma, Stephen M. Davis, Geoffrey A. Donnan, Sheila G. Crewther

**Affiliations:** ^1^Occupational Therapy, College of Science Health and Engineering, School of Allied Health, La Trobe University, Melbourne, VIC, Australia; ^2^Neurorehabilitation and Recovery, Stroke, The Florey Institute of Neuroscience and Mental Health, Melbourne, VIC, Australia; ^3^School of Psychology and Public Health, La Trobe University, Melbourne, VIC, Australia; ^4^School of Molecular Sciences, La Trobe University, Melbourne, VIC, Australia; ^5^School of Medicine, University of Tasmania, Hobart, TAS, Australia; ^6^Commonwealth Science and Industrial Research Organisation (CSIRO), Melbourne, VIC, Australia; ^7^The Florey Institute of Neuroscience and Mental Health, Parkville, VIC, Australia; ^8^Monash University, Clayton, VIC, Australia; ^9^The University of Melbourne, Parkville, VIC, Australia; ^10^Department of Medicine, Melbourne Brain Centre, Royal Melbourne Hospital, Melbourne, VIC, Australia

**Keywords:** ischemic stroke, proteomics, post-stroke depression, complement and coagulation, immunity and inflammation, stroke neurological recovery, blood biomarkers

## Abstract

Depression after stroke is a common occurrence, raising questions as to whether depression could be a long-term biological and immunological sequela of stroke. Early explanations for post-stroke depression (PSD) focused on the neuropsychological/psychosocial effects of stroke on mobility and quality of life. However, recent investigations have revealed imbalances of inflammatory cytokine levels in association with PSD, though to date, there is only one published proteomic pathway analysis testing this hypothesis. Thus, we examined the serum proteome of stroke patients (*n* = 44, mean age = 63.62 years) and correlated these with the Montgomery–Åsberg Depression Rating Scale (MADRS) scores at 3 months post-stroke. Overall, the patients presented with mild depression symptoms on the MADRS, M = 6.40 (SD = 7.42). A discovery approach utilizing label-free relative quantification was employed utilizing an LC-ESI–MS/MS coupled to a LTQ-Orbitrap Elite (Thermo-Scientific). Identified peptides were analyzed using the gene set enrichment approach on several different genomic databases that all indicated significant downregulation of the complement and coagulation systems with increasing MADRS scores. Complement and coagulation systems are traditionally thought to play a key role in the innate immune system and are established precursors to the adaptive immune system through pro-inflammatory cytokine signaling. Both systems are known to be globally affected after ischemic or hemorrhagic stroke. Thus, our results suggest that lowered complement expression in the periphery in conjunction with depressive symptoms post-stroke may be a biomarker for incomplete recovery of brain metabolic needs, homeostasis, and inflammation following ischemic stroke damage. Further proteomic investigations are now required to construct the temporal profile, leading from acute lesion damage to manifestation of depressive symptoms. Overall, the findings provide support for the involvement of inflammatory and immune mechanisms in PSD symptoms and further demonstrate the value and feasibility of the proteomic approach in stroke research.

## Introduction

Over 15 million people worldwide experience a stroke each year; 5 million of those events are fatal, and another 5 million people are left with a permanent disability (1). Previous epidemiological reviews have concluded that ~30% of stroke survivors are likely to experience post-stroke depression (PSD) (2, 3). Prevalence of depression is stroke survivors is reported to peak at 3 months post-stroke based on testing using the Diagnostic and Statistical Manual V [DSM-V (4)] (5). Studies have also characterized depression symptoms on the same criteria as early as 15 days and as late as 12 months post-stroke (5). Stroke patients with PSD show poorer functional and recovery outcomes compared to patients not suffering depression (6, 7). PSD is also found to contribute to poorer quality of life and increased mortality rates (1, 6), thus highlighting the need for patient care that extends beyond that of physical and cognitive rehabilitation. Investigation of biomarkers linked with underlying mechanisms has potential to guide the targeting of therapy, both prevention and treatment.

Currently, there is no consensus regarding the etiology of PSD with much debate as to the extent to which PSD stems from a purely biological origin and/or more likely incorporates elements of psychosocial response (7, 8). Following the biological argument for PSD progression, it was first proposed that the location of the stroke lesion could predict PSD presentation (9). This has been long debated without resolution (10). More recently, basal ganglia or frontal lobe lesions (7), white matter hyperintensities (11), and interruption of connecting pathways (12) have been linked to PSD. Furthermore, a recent review suggests that right hemisphere stroke predicts PSD incidence in the sub-acute, 1–6 months post-stroke period (13). Other explanations for correlations between PSD symptoms and stroke lesions have suggested that the overall balance of monoamines, such as serotonin, dopamine, and norepinephrine, are disrupted following cerebrovascular damage (14). These same amines have previously been associated with depression, consistent with a link between stroke and depressive symptoms (15).

Other reviews and meta-analyses have failed to find clear evidence for lesion location as a risk factor for PSD (3, 5). For instance, similar incidence rates of comorbid depression can be seen in other cerebrovascular diseases such as vascular dementia in the elderly where brain lesions are common (16, 17). Surprisingly, prevalence rates of depression are also similar in patients who have been subject to transient ischemic attacks (TIAs) (18–20) or carotid artery stenosis patients (21), where there is little to no presentation of lesions visible on computerized axial tomography or magnetic resonance imaging (MRI). In TIA especially, prevalence of PSD is comparable to stroke even at 12 months post-stroke (19). Thus, while currently detectable brain lesions may contribute to the etiology of PSD, they are unlikely to be the primary cause with evidence suggesting other pathogenesis involving immune disruption and circulating cytokines long after the stroke (22).

It is possible that PSD could be viewed as depression resulting first from damage caused by the initial ischemia and reperfusion injury (23) and exacerbated by psychosocial issues such as anxiety, loss of confidence, and apathy associated with even mild loss of mobility or functioning (24). In line with this view, the cytokine hypothesis proposes a role for pro-inflammatory cytokines such as tumor necrosis factor α, interleukin-1 (IL-1), IL-6, IL-8, and anti-inflammatory IL-10. This is consistent with evidence that pro-inflammatory cytokines have been found to be increased in both depressive mood (25, 26) and cerebrovascular events (8, 16, 27, 28). Pro-inflammatory cytokine levels are elevated during and long after the therapeutic window of typical tissue plasminogen activator treatment (tPA; <4.5 h) (16, 29, 30). Recent immunoassay research has established that cytokine levels often remained significantly elevated at 1 year post-stroke, leading the authors to suggest that the homeostatic balance of pro- and anti-inflammatory cytokines may be disrupted in the long term (31). Elevated pro-inflammatory cytokine expression is also likely to influence glucocorticoid resistance and sensitivity, which is reported to lead to overactivation of the hypothalamus–pituitary–adrenal (HPA) axis, thereby inducing depressive mood (32–34). Furthermore, persistently high concentrations of pro-inflammatory cytokine levels may provide functional markers for long-term impaired immunological responses as the body attempts to resolve the lesion-induced neurodegeneration.

Given the multifaceted nature of PSD, it is difficult to ascertain the underlying molecular basis of stroke damage associated with depressive symptoms at the 3-month peak prevalence period in human patients. In recent years, proteomics has provided a powerful platform for pathology research. Downstream from transcriptomic processes, the proteome describes the totality of proteins that can be produced by the organism’s genome. The complexity of the proteome accounts for the biological functioning of an organism and as such includes not only the primary function of the protein itself but also how it fits into the biological environment that is further determined by protein–protein interactions, posttranslational modifications (35), and protein degradation. With the addition of alternative splicing to these processes, the 25,000 genes in the human genome can theoretically encode up to 1,000,000 different proteins (36). To this end, proteomics approaches coupled with bioinformatics have proven useful in identifying potential therapeutic biomarkers in pathologies including cancer (37) and cardiovascular disease (36). In the case of proteomic disease research, biomarkers refer to a subset of proteins that are upregulated, downregulated, activated, or deactivated and are detectable using a particular methodology in a disease phenotype.

The proteomic composition of a biological organism can reflect not only rapid changes in response to external challenges (38, 39) but also can be studied to understand disease progression and biological changes in the long term (40, 41). In stroke, the damage caused by the event would be expected to show both acute and later chronic consequences. A discovery proteomics approach utilizing a mass spectrometer with high dynamic range in order to assay as much of the human proteome as possible is therefore warranted in the context of proteome complexity and the multifactorial nature of PSD etiology. Previous studies adopting this approach have been successful in providing new insights and support for the interpretation of molecular biological mechanisms following a stroke (42). The majority of these studies focused on revealing biomarkers that might be useful aids to rapid diagnosis, for example, in the case of differentiation between hemorrhagic stroke versus ischemic stroke (43) and ischemic stroke versus healthy controls (44). Research has also been successful in characterizing the treatment effects of tPA (45) and electroacupuncture (46) on the human proteome, demonstrating that proteomics approaches can objectively characterize the molecular changes associated with certain treatments.

A review of the literature up to December 2015 revealed only a single published study examining the proteomic profile of PSD. Isobaric tagging for relative and absolute quantification (iTRAQ) was employed by Zhan and colleagues (47) to compare the ethylenediaminetetraacetic acid (EDTA) anticoagulated blood of patients with stroke and PSD, stroke without PSD, and healthy controls. The iTRAQ approach involves isobaric tagging of tryptic digested samples based on phenotypes of interest for comparison through commercially available iTRAQ reagents and thus facilitates proteome to proteome comparisons of disease versus control groups (48). This is conducted by introducing a pooled mixture of all the samples to the mass spectrometer, whereby relative quantitation can be assessed by comparing differences in the peak intensity between labeled peptides (49), an approach well suited for investigations of disease phenotypes. The Database for Annotation, Visualization and Integrated Discovery (DAVID) bioinformatics tool for gene enrichment revealed that of the peptides that statistically differentiated stroke patients with and without PSD, only the complement and coagulation cascade pathway accounted for the clustering of identified proteins. Given that this research was conducted on patients at 1 month post-stroke, it was suggested that these findings indicate a homeostatic imbalance of pro-inflammatory and anti-inflammatory processes in PSD (47). These findings are in line with the principles of the cytokine hypothesis of PSD, albeit complement pathways are more reliably identifiable in blood as biomarkers of immune disruption (50, 51). Zhan and colleagues (47) also further examined the top protein candidates by protein immunoblotting of apolipoproteins A IV (ApoA-4) and C-II (ApoC-2), C-reactive protein (CRP), gelsolin, haptoglobin, and leucine-rich glycoprotein (LRG). All of these proteins were significantly altered in PSD patients compared to stroke without depression. As there are limited data on the biological processes directly involved in PSD, Zhan et al.’s (47) study represents a successful first step in applying proteomics approaches to PSD, a disorder that has been previously difficult to characterize in molecular terms.

As the molecular basis of depression after stroke is not well understood, we aimed to take a discovery approach to identifying molecular pathways impacted by depression in stroke survivors. We sought to use a proteomics technique that is capable of identifying and quantifying a large number of biological entities. Our research aim was to identify and quantify peptides to be used to understand the biological mechanisms associated with PSD symptoms. Thus, a data-driven discovery proteomics methodology was used to investigate the biological mechanisms associated with depression etiology 3 months post-stroke. Given the recent finding of Zhan et al. (47), we also sought to investigate whether this finding could be replicated using a different proteomics and analytic approach to that previously reported, in order to strengthen the validity of the proteomic profile of PSD. Our analysis was conducted at 3 months post-stroke.

First, a label-free quantitation (LFQ) technique was employed to examine the blood samples of stroke patients. The LFQ technically differs from the iTRAQ approach in that the protein samples are not pooled or tagged for analysis, and each sample generates its own proteomic profile (52). The relationship between protein expression and various clinical measurements can then be explored instead of comparisons being planned *a priori*, thus conforming to a discovery approach better suiting our aim. Furthermore, it is debatable that iTRAQ provides better quantitation than LFQ approaches, with LFQ shown to perform similarly (53) or more accurately (54) than iTRAQ approaches.

Second, blood serum was chosen compared to EDTA anticoagulated plasma. To date, there has been little or no research published comparing the differences between serum and plasma blood samples in stroke proteomics. EDTA has been shown to be the least proteolytically active of the plasma samples and has previously been shown to reveal low abundance proteins in healthy patients (55). However, the proteomic and secretomic profile of serum may be more suitable for profiling of coagulation and complement systems (56). Furthermore, in a small batch optimization analysis of these bloods, we found overall increased expression of coagulation and complement in serum compared to EDTA, as expected (57).

Third, biological pathways and mechanisms were ascertained using the gene set enrichment analysis (GSEA) statistical and bioinformatics tool. This tool compares the current protein expression data from our study to those from the Molecular Signatures Database (MSIGDB) (58). The approach of DAVID considers gene annotation clustering after statistical procedures have identified significant differences in protein expression (59). However, the GSEA approach develops an enriched gene set from the original expression data and compares them to sets from the databases on MSIGDB (58). Using pathway analysis affords greater explanatory power in highlighting relationships between gene sets and phenotypes that might go unnoticed in a comparison of individual proteins. While this is inherently a technique for genes and genomic profiling, the statistical approach of database list ranking in GSEA has been demonstrated to be applicable to mass spectrometry (MS)-based proteomics (60–62). The databases cannot yet interpret the full complexity of the molecular proteome as they do not consider protein/protein interactions and post-translational modifications. However, despite these limitations, GSEA represents one of the best tools currently available for understanding cellular pathways (63). Ultimately, we aim to show that the proteomics approach is a reliable and viable method informing our understanding of PSD and stroke pathophysiology.

## Subjects and Methods

### Subjects

A subset of 44 stroke patients (30 females and 14 males) were obtained consecutively and prospectively from the START_PrePARE (*ST*roke im*A*ging p*R*evention and *T*reatment_*Pre*diction and *P*revention to *A*chieve optimal *R*ecovery *E*ndpoints) cohort, a longitudinal stroke cohort study with advanced clinical and neuroimaging data conducted in Australia (64). The participants were recruited following a first ischemic stroke and were over 18 years of age. A diagnosis of ischemic stroke was determined by an experienced neurologist using clinical assessments supplemented with computerized tomography (CT) or MRI. Further information on inclusion and exclusion criteria for this study can be found in protocol papers for START_PrePARE (64) and START_EXTEND (65). Patients included were selected consecutively from the prospectively collected START_PrePARE cohort as patients were being recruited. Patients were not excluded if they had a prior history of depression, as our focus was to identify biological factors associated with the presence of depression in stroke survivors at a particular point in time, irrespective of whether depression was present prior to or post-stroke (64). Inclusion of patients with prior history of depression was also important to improve ecological validity and permit generalization of findings to clinical populations, given prior history of depression in stroke patients.

### Design

The data presented in this manuscript were obtained primarily at 3 months post-stroke (±7 days), when clinical data and blood were collected. Ethics was approved by the Human Research Ethics Committee of Austin Hospital, Heidelberg (HREC code: H2010/03588), and relevant university and hospital sites. Further details on study design are provided in related protocol papers for START_PrePARE (64) and START_EXTEND (65).

### Clinical Assessments

The National Institute of Health Stroke Scale (NIHSS) was administered by a trained neurologist or health-care professional. This measure is designed to be a test of patient neurological status and correlates highly with stroke severity (66). Prior history of depression was obtained using the two-item Patient Health Questionnaire (PHQ-2) (67) at 3–7 (±1) days post-stroke and again at 12 months (±7 days) post-stroke. The PHQ-2 is a brief screening tool that employs the first two questions of the nine-item Patient Health Questionnaire (PHQ-9) (68) and shares high correlation, interrater reliability, and internal consistency with the PHQ-9 (69).

Depressive symptoms were assessed at 3 months post-stroke using the Montgomery–Åsberg Depression Rating Scale (MADRS). The MADRS is a validated measure in clinical depression research (70, 71), and was delivered using the structured interview guide (SIGMA) (72). This is a standardized interview format for the MADRS, providing the clinician with more versatility to probe the circumstances surrounding depression symptomatology compared to a self-report method. The SIGMA format is reported to have higher reliability than the self-report MADRS (72). The MADRS_SIGMA was selected as it is a standardized and validated observer-rating of depression at a point in time (72). It was administered by a health-care professional (stroke nurse, occupational therapist, or doctor) specifically trained in the administration of this assessment. Assessors used SIGMAs, detailed protocol manuals, and training videos to enhance standardization in the administration of the tool. Non-verbal supports were available to patients who were aphasic. Higher scores on the MADRS indicate more depressive symptoms.

Global cognitive impairment was screened using the Montreal Cognitive Assessment (MoCA), a validated screening measure of cognitive impairment in dementia and post-stroke recovery (73). While positive scores on the MoCA indicate optimal cognitive functioning, an adjusted cutoff score of <23 in stroke populations suggests cognitive impairment (74). The modified Rankin Scale (mRS) was used to measure functional disability. It is an interview based measure, with lower scores indicating lesser levels of observable functional impairment in daily life (75). All assessments were administered by a health-care professional, specifically trained in the administration of these assessments. Background details on age, gender, subtype of acute ischemic stroke, and thrombolysis were also obtained.

### Blood Collection and Serum Separation

Blood samples were obtained by venipuncture at the 3-month follow-up assessment. All samples were collected in plastic serum-separating tube (SST) vacutainers and were allowed to clot at ambient temperate for 30 min. The tubes were then centrifuged at 1100–1300 *g* at room temperature, and the resulting serum was aliquoted into Eppendorf 4 × 2.0 mL tubes and immediately stored at −80°C. Upon moving blood samples from hospital sites to the central laboratory, temperature was kept at −20°C prior to transfer into a −80°C freezer.

### Sample Preprocessing and Trypsination

Ten microliters of serum from each patient was first stabilized in 100 μL of 8M urea pH = 8.3 and stored at −80°C until used. For proteomic analysis, the stabilized samples were processed as follows: 20 μL protein solution was added to 90 μL of 8M urea pH = 8.3 and reduced for 5 h with 1 μL of 200 mM tris(2-carboxyethyl)phosphine (TCEP). After this, samples were alkylated for 1 h at 25°C in the dark with 4 μL of 1M iodoacetamide (IAA). In sample, digests were performed overnight (37°C) by addition of 1 μg of trypsin (Promega, Madison, WI, USA) and 900 μL of 50 mM Tris pH = 8.3, followed by a second digestion step with 1 μg trypsin and an additional incubation of 4 h at 37°C. Two hundred microliters of the digested solution were collected and dried by SpeedVac centrifugation. The digested proteins were resuspended in 100 μL of 1% (v/v) formic acid and centrifuged at 14,000 rpm for 2 min. The solid-phase extraction was performed with Empore reversed-phase extraction disks (SDB-XC reversed-phase material, 3M) according to Ishihama et al. (76) with the following modifications: the membrane was conditioned with 50 μL of 80% (v/v) acetonitrile, 0.1% (w/v) trifluroacetic acid, and then washed with 50 μL of 0.1% trifluroacetic acid before the tryptic peptides were bound to the membrane. The bound peptides were eluted by 50 μL 80% (v/v) acetonitrile, 0.1% (w/v) trifluroacetic acid, and dried in a SpeedVac centrifuge.

### Mass Spectrometry

Tryptic peptides reconstituted in 0.1% formic acid and 2% acetonitrile (buffer A) were analyzed by LC-ESI–MS/MS on a LTQ-Orbitrap Elite (*Thermo-Fisher Scientific*). Peptides were loaded onto a trap column (C18 PepMap 100 μm i.d. × 2 cm trapping column, *Thermo-Fisher Scientific*) at 5 μL/min for 6 min before switching the precolumn in line with the analytical column (Easy-Spray 75 μm i.d. × 50 cm, *Thermo-Fisher Scientific*). The separation of peptides was performed at 250 nL/min using a linear acetonitrile gradient of buffer A and buffer B (0.1% formic acid and 80% acetonitrile), starting from 5% buffer B to 60% over 300 min. This final separation step is equivalent to fractionation and was conducted in order to avoid potential biases and increased sample variability due to depletion. Although it is impossible to eliminate dynamic range issues in serum samples, this technique greatly increases the dynamic range detectable in our experiment.

Data were collected in data-dependent acquisition mode using *m*/*z* 300–1500 as MS scan range; CID MS/MS spectra were collected for the 20 most intense ions. Dynamic exclusion parameters were set as follows: repeat count 1, duration 90 s, and the exclusion list size was set at 500 with early expiration disabled. Other instrument parameters for the Orbitrap were the following: MS scan at 120,000 resolution, maximum injection time 150 ms, AGC target 1 × 106, and CID at 35% energy for a maximum injection time of 150 ms with AGT target of 5000. The Orbitrap Elite was operated in dual analyzer mode with the Orbitrap analyzer being used for MS and the linear trap being used for MS/MS. This procedure was performed on two technical replicates. The samples were then analyzed using the in-house Mascot server for protein identification. MaxQuant (Max-Planck Institute for Biochemistry, Martinsried, Germany) was used to obtain the relative quantification of identified proteins in the samples. Relative intensity or quantification is a measurement of peak height in a single sample that is compared to the same measurement in other samples. The absolute concentrations of the proteins in the sample are not known and require other methodologies to obtain, and thus, relative protein expression data cannot be generalized to other protein assays. Absolute quantification is possible on a mass spectrometer, but requires prior knowledge of target proteins and extensive methodologies.

### Protein Identification and Label-Free Quantitation

Identification and LFQ of obtained spectra across all 44 samples was performed using MaxQuant version 1.4.1.2 to obtain identified proteins (77). All raw data and complete details of MaxQuant parameters and result files have been deposited in ProteomeXChange and are available with accession number PDX003494. Identification of peptides and proteins was performed internally by MaxQuant using the Andromeda (77) search engine to search against all reviewed and unreviewed human proteins in the Uniprot database (August 2013; 133,798 entries in total). Common contaminants and decoys (reversed sequences) were included automatically by Andromeda. Prior to searching, MS/MS spectra were filtered according to MaxQuant default settings for ion trap MS/MS spectra by retaining only the top eight peaks per 100 Da. Main search, precursor mass tolerance was set to 4.5 ppm, and MS/MS tolerance to 0.5 Da. Carbamidomethylation of cysteines was set as a fixed modification, and *N*-term acetylation and oxidation of methionine were included as variable modifications. Up to two missed cleavages were allowed, and peptides were required to be at least seven amino acids in length. False discovery rate (FDR) cutoffs for both peptides and proteins in the database search were set to 1%. Both unique and razor peptides were used for quantitation with a minimum of two peptides including at least one unique peptide required to calculate a protein quantitative value. The “match between runs” setting in MaxQuant was used to transfer peptide identifications from one run to another on the basis of matching retention time and mass-to-charge ratio.

### Data Preprocessing and Analysis

The initial output from MaxQuant consisted of 515 protein groups. After removal of contaminants, the list was shortened to 475 protein groups. The LFQ signal intensity was log_2_ normalized to account for naturally skewed intensity values (78) and averaged over technical replicates. The discovery approach here employs relative quantification instead of absolute, and as such, it was better suited to examine a collection of genes/proteins as ontologies of biological structures or pathway processes to extract biological significance from the proteomics data and understand the systems involved in depression symptoms post-stroke (63). As relative protein expression is limited to comparison within the study or similar MS-based studies, pathway analysis provides greater explanatory power than traditional statistical biomarker approaches. Thus, the resulting protein group expression data were reduced to gene names then paired with the continuous MADRS scores to prepare for GSEA (Broad Institute, MIT). GSEA allows for a robust comparison of continuous phenotypes to gene expression with the selection of the “Pearson” metric. All analyses in GSEA were conducted with the GENE_SYMBOL chip and default number of permutations (1000) on full gene sets from Hallmark, Gene Ontology (GO), the Kyoto Encyclopedia of Genes and Genomes (KEGG), Biocarta, and Reactome Positional and Immunologic Signatures, acquired from MSIGDB v5.00. The Hallmark database is recommended as an entry to GSEA analysis, as it collects gene sets that represent well-defined biological states and processes with expression scores computed from many existing gene sets to reduce noise and redundancy, acting as the searchable “meta-analysis” of gene sets. GO is the earliest but most up-to-date functional gene annotation database and encompasses the largest variety of annotations under three headings: biological processes, molecular function, and cellular component (79). KEGG (80), Biocarta (81), and Reactome (82) gene sets are curated from external databases, with each database representing different approaches to compiling, such as genome sequencing, microarray profiling, and computational methods, to build complex networks. Positional gene sets represent the locations of genes on chromosomes and cytogenic bands (83), while the Immunologic Signatures database is a collection of immune responses curated from separate microarray studies. Included gene sets were no smaller than 15 and no larger than 500. As per recommendation by the GSEA program for discovery experiments, a FDR of 25% was deemed acceptable for statistical significance of enriched gene sets, where there is a 75% chance of rejecting a false positive (58). A nominal *p* (nom-*p*) value based on the statistical significance of each individual database is also included for reference. This statistic is not adjusted for multiple testing, whereas the FDR value is.

An enrichment score is a Kolmogorov–Smirnov-like ranking statistic that reflects the degree to which a set is overrepresented at the top (positive enrichment) or bottom (negative enrichment) of the list when compared to another list (58). For example, when adjusted for correlation with increasing score on a clinical scale, such as the MADRS, positive enrichment suggests biological upregulation of a given gene list, while negative enrichment suggests downregulation of a gene list with increasing presentation of depression symptoms. The normalized enrichment score (NES) is the primary statistic used for evaluating and comparing gene sets and is understood as (58):

$$\text{NES}=\frac{\text{actual Enrichment Score}}{\mathrm{mean}(\text{Enrichment Scores against all permutations of the dataset})}$$

This analysis yields the “leading edge subset” that refers to the cluster of proteins that contribute most to the enrichment score and can be interpreted as the genes that are most likely to affect change in complex pathway function.

To minimize the possible confound of prior history of depression on the analysis, the appropriate *T*-test or non-parametric (Mann–Whitney) comparisons were conducted and compared with group comparisons with Monte Carlo simulations of the MADRS scores in subgroups with and without prior history of depression.

## Results

### Patient Demographics and Clinical Status

The average age of ischemic stroke patients in the sample was 63.62 years, range 34–87 (SD = 13.52), within the range of previous epidemiological findings, although younger than the mean age of 74.40 years (84). Thirty were males and 14 females. Based on the TOAST classification (85), 11 patients had large artery atherosclerosis, 7 had cardioembolism, and 11 had small vessel occlusion. The remaining 15 patients were unclassified. Nine patients were administered thrombolytic tPA within the 4.5-h time window and two were unknown. Patient demographics and NIHSS score at time of recruitment (i.e., baseline M = 1.39, SD = 1.26 days post-stroke), and clinical characteristics at 3–7 days and at 3 months post-stroke are presented in Table 1.

**Table 1:** **Patient characteristics at baseline, 3–7 days, and 3 months post-stroke**.

Patient characteristic	Baseline^a^	3–7 days (±1 day) post-stroke	3 months post-stroke (±7 days)
Marital status, *n* (%)			
Single	2 (4.5)
Married	30 (68.2)
Widowed	4 (9.1)
Separated or divorced	6 (13.6)
Other	2 (4.5)
Working status, *n* (%)			
Employed	25 (56.8)		14 (32)
Given up work	–		7 (16)
Returning to work	–		4 (9)
Homemaker	1 (2.3)		1 (2)
Retired	18 (40.9)		18 (41)
Living situation, *n* (%)			
Home alone	9 (20)		8 (18.2)
Home with others	34 (77)		34 (77.3)
Retirement home	1 (2)		1 (2.3)
High level care nursing home	–		1 (2.3)
Schooling completed (years), mean (SD)	11.81 (3.66)		
NIHSS (1–42), mean (SD)	3.84 (4.03)	1.68 (0.33)	0.36 (0.12)
mRS (0–6), median (IQR)	0.00 (0.00)^b^	–	1.00 (2.00)
MoCA (0–30), mean (SD)		25.70 (0.43)	26.70 (0.45)
MADRS (0–60), mean (SD)		7.16 (5.75)	6.40 (7.42)

*^a^Baseline measurements were taken at M = 1.39, SD = 1.26 days post-stroke, i.e., at time of recruitment to the study*.

*^b^This mRS score was taken at recruitment and assesses pre-stroke functioning*.

*NIHSS, National Institute of Health Stroke Scale; 1–42; <5 suggests mild stroke severity, with 42 being most impaired; mRS, modified Rankin Scale; range: 0–6, with a score of 1 indicating no significant disability, despite symptoms, and able to carry out all usual duties and activities; MoCA, Montreal Cognitive Assessment; range: 0–30, <23 mild global cognitive impairment; MADRS, Montgomery–Åsberg Depression Rating Scale; 0–60, >6 suggests presence of depressive symptoms, >17 indicates experience of a major depressive episode within 2 weeks*.

The average MADRS score for the sample was 6.40 (SD = 7.42, 95% CI = [4.17, 8.63], range 0–26). Thirteen (29.55%) patients had a score of >6 but <18, indicating that this sample mostly had mild depressive symptoms. Only 4 (9.09%) patients had scores within the severe symptoms range. However, a person scoring 12 in major depressive disorder or 8 post-stroke on the MADRS can be considered for treatment (86). The sample presented with mild stroke severity at 3 months, with a median NIHSS score of 0 and range 0–5. Similarly, scores on the MoCA and mRS suggest that this sample had relatively few cognitive problems and had recovered in daily activities at 3 months from the stroke. A correlation analysis of the MADRS with these clinical measures and patient age revealed no significant results at 3 months post-stroke.

Screening on the PHQ-2 revealed that 11 of the 44 patients had a prior history of depression. As it was possible that patients with a pre-stroke history of depression may be predisposed to more severe PSD symptoms, a Mann–Whitney comparison was conducted to test heterogeneity of MADRS scores between those with and without prior history of depression. Preliminary analysis suggested that the distributions of both groups were similar, and thus, the combined median metric is used (87). After running a Monte Carlo simulation for 1,000,000 samples at 99% CI to account for low sample sizes, it was found that patients with a previous history of depression (Mdn = 5.00) did not significantly differ from patients without any history (Mdn = 2.50), *U* = 165.00, *z* = −0.591, Monte Carlo *p* = 0.565 [99% CI = (0.563, 0.566)]. Following this validation, it was possible to continue with the available range of MADRS scores without having to categorize based on prior history of depression.

### Gene Set Enrichment Analysis

Peptides (*n* = 475) from the proteomic analysis were analyzed to reveal underlying molecular pathways and gene ontologies associated with depressive symptoms. Five different data sets (Hallmark, GO, KEGG, Biocarta, and Reactome) were interrogated to identify enriched data sets. Multiple databases were used in our discovery approach to not only explore a comprehensive range of possible pathways (given the fact that each database is constructed differently) but also to identify commonalities across databases, thus strengthening the robustness and generalizability of our findings.

Of the gene set databases that were entered in GSEA (Table 2), Positional and Immunologic Signatures did not return enriched sets. With the exception of GO, all databases showed significantly enriched gene sets pertaining to complement cascade activation or general immune upregulation/downregulation. All sets were negatively enriched when compared with increasing MADRS score, suggesting significant protein downregulation with increasing level of depressive symptoms. There was also no clear enrichment pattern that would associate individual genes with MADRS scores. However, there are slight variations in set size and top gene contributors that reflect the differences in the compiling of the databases itself. Thus, although the statistics and bioinformatics analysis show that these pathways are associated with depression, biological interpretation is dependent upon further information from the individual databases. Positional gene sets collect data about chromosomal cytogenic band positioning of the genes involved, while Immunological Signatures collect published examples of specific immune activity against an immune challenge such as dendritic cell activity in human immunodeficiency virus.

**Table 2:** **Size of gene set databases searched in GSEA and corresponding number of enriched sets adjusted for FDR**.

Gene set database	Gene sets	Enriched gene sets	Significantly enriched gene sets (FDR <25%)
Hallmark	50	3	2
Gene Ontology	1454	25	0
KEGG	186	2	2
Biocarta	217	1	1
Reactome	674	7	3
Positional	326	0	0
Immunologic Signatures	1910	0	0

*These gene sets are publicly available at http://software.broadinstitute.org/gsea/msigdb/collections.jsp*.

### Hallmark

As the Hallmark gene sets are computed from a collection of similar biological processes, this set provides strong support for the negative enrichment of this set and associated gene expressions (Table 3). A negatively skewed NES for both coagulation and complement Hallmark gene sets correlated against increasing MADRS scores indicates that there is an overexpression of downregulated genes in these sets. These genes can be seen in the leading edge subset (highlighted in bold), while the other genes comprise the structure of the set and are important in affirming the construct validity of the obtained sets, which do not significantly contribute to the overall magnitude and direction of the enrichment score.

**Table 3:** **Significantly enriched gene sets and individual gene contributors from the hallmark database**.

Name	NES	Individual gene contributors	FDR	Nom *p*
Coagulation	−1.268	**COMP PROC MBL2 TF C8B C9 APOC1 F2 PROZ FN1 PLG GSN MST1 F11**	0.218	0.157
		GP1BA KLKB1 CFI APOC3 C2 ITIH1SPARC HRG CLU CPB2 F10 SERPINC1 PF4 SERPING1 F13B F12 APOA1 A2M C1R FGA APOC2 VWF C1QA CFH PROS1 SERPINA1 CFB CPN1 ANG C8G C1S C8A C3		
Complement	−1.662	**CP S100A12 C9 C1QC APOC1 F2 FN1 PLG**	0.157	0.013
		LTF GP1BA KLKB1 C2 ITIH1 CLU CA2 F10 SERPINC1 F5 SERPING1 C1R C4BPB C1QA CFH SERPINA1 CFB APOA4 ANG C1S C3		

*The genes in bold represent the leading edge subset of genes (see Subjects and Methods for overview) that contribute most to the enrichment score of the set*.

*NES, normalized enrichment score; FDR, false discovery rate; Nom *p*, nominal *p*-value*.

### Kyoto Encyclopedia of Genes

Coagulation and complement cascades and systemic lupus erythematous (SLE) were implicated in the KEGG database (Table 4). In this database, the SLE set is defined by the antigen-activated complement pathway, demonstrating the characteristic molecular cascades of immune dysfunction. Even though SLE is a significant risk factor of ischemic stroke (88), as seen from the leading edge (Table 4), it is more likely that this set was merely expressing statistical enrichment of a gene set that is largely comprised of complement. Central to all complement pathways, complement component 3 (C3) is one of the main activators and mediators of this pathway and was found to contribute highly to the negative enrichment score.

**Table 4:** **Significantly enriched gene sets and individual gene contributors from the KEGG database**.

Name	NES	Individual gene contributors	FDR	Nom *p*
Complement and coagulation cascades	−1.377	**C5 C4B C8G C1S KNG1 C8A C3 PROC MBL2 C8B C9 C1QC F2 C4BPA C1QB SERPINF2 PLG C6 F11**	0.083	0.070
		FGB SERPINA5 KLKB1 CFI C2 SERPIND1 CPB2 F10 SERPINC1 F5 MASP1 SERPING1 F13B F12 A2M C1R FGA VWF C4BPB C1QA CFH C4A PROS1 SERPINA1 CFB C7		
Systemic lupus erythematous	−1.731	**CP S100A12 C9 C1QC APOC1 F2 FN1 PLG**	0.019	0.016
		C7 C5 C4B C8G C1S C8A C3 C8B C9 C1QC C1QB C6		

*The genes in bold represent the leading edge subset of genes that contribute most to the enrichment score of the set*.

### Biocarta

The set size of Biocarta complement was only 17, with the majority of genes contributing to the various complement pathways and formation of the membrane attack complex (MAC) that is involved in attacking target cells. This gene list suggests that both mannan-binding lectin 2 (MBL2) and complement component 1 (C1Q), precursors of both the lectin and classical pathways, are downregulated in association with depressive symptoms (Table 5). C3 is central to the entire cascade as its activation is required for both lectin and classical pathways and attraction of the adaptive immune system.

**Table 5:** **Significantly enriched gene sets and individual gene contributors from the Biocarta database**.

Name	NES	Individual gene contributors	FDR	Nom *p*
Complement	−1.626	**CFB C7 C5 C4B C1S C8A C3 MBL2 C9 C1QC C1QB C6**	0.029	0.029
		C2 MASP1 C1R C1QA C4A		

*The genes in bold represent the leading edge subset of genes that contribute most to the enrichment score of the set*.

### Reactome

**Figure 1:**
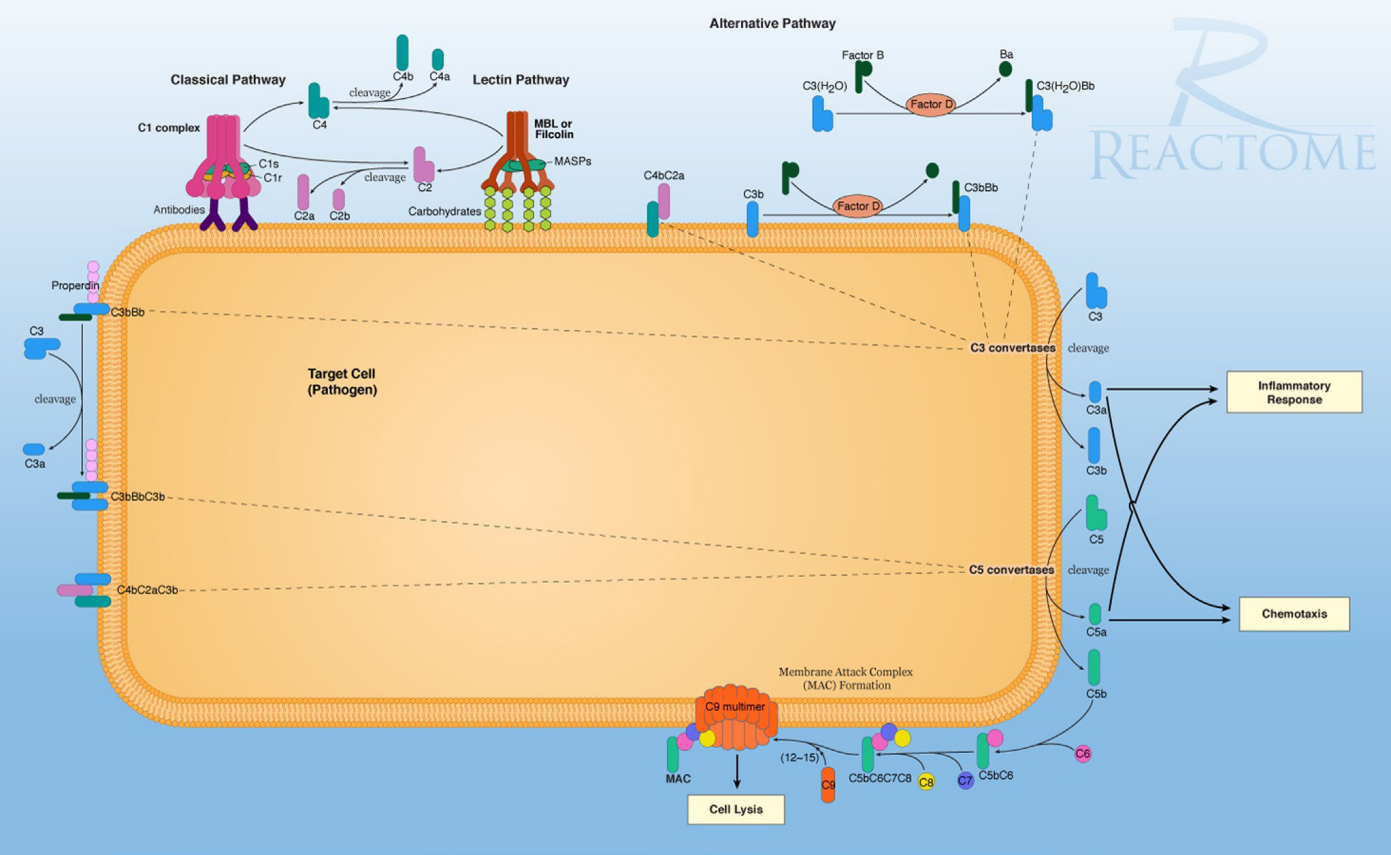
**The reactome pathway diagram, demonstrating the primary signaling and functional molecular pathways of the complement system on a target cell**. In this diagram, multiple activation pathways are shown, eventually effecting cell lysis and breakdown of the target cell. This also shows secondary signaling pathways for C3a signaling for pro-inflammation and cytokine activity with C5a-promoting chemotaxic recruitment of inflammatory cells. Retrieved from: http://www.reactome.org/figures/Pathway_Illustrations/Complement_Cascade_72_oicr.png; reused under the Creative Commons license.

The Reactome database implicated three gene sets with significant negative enrichment in association with MADRS scores, all with similar individual gene contributions (Table 6). This database consists of a large pathway map of which complement cascades are categorized under the innate immune system. The results for the complement pathways were similar to those obtained previously; both classical and lectin pathways are downregulated, and MAC activation genes, such as C9 and C6, are underrepresented. These genes are essential to the overall function of the complement system (Figure 1).

**Table 6:** **Significantly enriched gene sets and individual gene contributors from the Reactome database**.

Name	NES	Individual gene contributors	FDR	Nom *p*
Immune system	−1.651	**C7 C5 C8G C1S CARD9 C8A C3 ICAM1 MBL2 C8B S100A12 C9 C1QC C4BPA C1QB C6 LBP**	0.075	0.029
		CRP CFI C2 MASP1 SAA1 SELL CD14 C4BPB C1QA CFH C4A PROS1 B2M CFB		
Innate immune system	−1.639	**C7 C5 C8G C1S CARD9 C8A C3 MBL2 C8B S100A12 C9 C1QC C4BPA C1QB C6 LBP**	0.039	0.018
		CRP CFI C2 MASP1 SAA1 CD14 C4BPB C1QA CFH C4A PROS1 CFB		
Complement	−1.334	**C7 C5 C8G C1S C8A C3 MBL2 C8B C9 C1QC C4BPA C1QB C6**	0.194	0.126
		CRP CFI C2 MASP1 C4BPB C1QA CFH C4A PROS1 CFB		

*The genes in bold represent the leading edge subset of genes that contribute most to the enrichment score of the set*.

## Discussion

Depression after stroke is a common occurrence, negatively impacting functional outcome, response to rehabilitation, and quality of life. Utilizing a discovery proteomics approach, this study was aimed to find biological associates of depressive symptoms (MADRS scores) post-stroke in human serum. This was achieved by employing a label-free proteomics workflow, utilizing an LTQ Orbitrap with high dynamic range to maximize the range of protein identification. Statistical and bioinformatics analysis was completed on the GSEA platform on a wide range of curated gene set databases. This approach revealed consensus among well-developed databases for decreased gene expression that was associated with the complement pathways and partial support for precursors of the coagulation pathway.

The most important findings of this study are downregulation of coagulation and complement cascades in serum bloods from stroke patients with increasing level of MADRS-defined depressive symptoms, as depicted by Hallmark, KEGG, Reactome, and Biocarta databases. Most PSD studies examine a severe depression phenotype. However, we have shown here that even patients who have mild depressive symptoms and have recovered well can still exhibit biological changes that can be characterized by a proteomics approach. Furthermore, even though the analytical approach was different, our findings provide additional support for those obtained by Zhan et al. (47). Our findings also support the dysregulation of the coagulation and complement cascade pathway that was identified in Zhan et al. (47), in serum as opposed to EDTA blood samples. The contributing differences in individual gene expression are likely due to a number of factors, such as stroke timeline, study design, and statistical approaches, but most importantly, the use of serum contrasted with EDTA for blood preparation. Considering these methodological differences between the two studies, finding similar results only provides further confirmation for the proteomic profile of stroke and strengthens biological understanding of PSD.

From a molecular standpoint, the primary function of the complement system can be summarized as formation of the MAC on target cells, while secondary functions include signaling for toll-like receptor 4 (TLR4)-mediated inflammatory response (89), leading to increased pro-inflammatory cytokine levels (90). Complement has been traditionally viewed as a versatile system, conforming to three distinct activation pathways, leading to similar outcomes (91). Of the leading edge subsets of the databases searched, it is clear that a majority of the molecular determinants of the complement system are downregulated in relation to mild depressive symptoms. Of note, complement component 1q (C1q) and complement component 3 (C3) are both integral to the activation of this system, while downstream products that contribute to inflammatory signaling and formation on the MAC (Figure 1) are all downregulated. This may indicate perturbation in brain homeostasis following stroke, characterized by an ongoing state of lowered resistance to oxidative stress (92) and immune-related changes (93) in maintaining depression symptoms post-stroke. This is further implied by the Reactome database analysis, demonstrating negative enrichment of calcium-binding protein A12 (S100A12) and lipopolysaccharide-binding protein (LBP), attributed to innate immune functioning. S100A12 is an established cytoskeletal protein involved in the signaling of neutrophil response and a candidate biomarker of inflammation (94).

Coagulation is a key process in vascular diseases, especially in ischemic stroke where coagulation factors have been identified as pre-stroke risk factors (95). This process is heavily involved in the stroke itself and treatment with tPA (96, 97). Coagulation is an ongoing process in serum and deficiencies of several proteins, such as protein Z (PROZ) (98), fibronectin 1 (FN1) (97), and gelsolin (GSN) (99), have been identified to be involved in cerebrovascular and cardiovascular diseases. The overlap of genes between coagulation and complement sets is expected as the coagulation processes have been identified to cleave into the central components of the complement system (100). The inclusion of both complement and coagulation cascades into one gene set in KEGG compared to the presentation of two separate sets in Hallmark illustrates a previous debate as to whether the pathways should be considered separately (101, 102). However, further studies have shown that while complement deficiencies alone do not increase bleeding frequency and coagulation deficiencies alone do not impair immune responses, the functions of the two pathways are linked and indeed in the case of innate immunity (103, 104). It has been suggested that coagulation and complement are involved in a feedback loop, where complement activation increases platelet activation area on the target cell that in turn augments complement activity (105). Coagulation has independently been shown to be involved in the process of cell death and is associated with activation of the kinin–kallikrein pathway and facilitates defensive inflammatory responses by enhancing leukocyte activity (106). Finally, binding platelets also have the ability to enhance neutrophil activity *via* TLR4 signaling, a process that is vital to phagocytosis (105).

Although a robust association between relative presence of depressive symptoms and downregulation of complement and coagulation pathway was found in our necessarily small sample of mild stroke survivors living in the community, our findings need to be interpreted with care, especially as some patients had a prior history of depression. We did use a group comparison utilizing Monte Carlo simulations that indicated that prior depression did not significantly impact our findings. Due to the discovery approach and the patient’s characteristics of this sample, the generalizability of the findings is limited to survivors with mild stroke severity.

### Complement Post-Stroke: From Acute Cell Death to Immunodepression and Depression

Cell death in the brain is the inevitable consequence of any stroke damage. In stroke, the first wave of cell death occurs as a result of hypoxia. This releases damage-associated molecular patterns (DAMPs) that can begin and perpetuate apoptotic and necrotic cell death cascades in neighboring neurons. Such molecules can include, but are not limited to, intracellular adenosine triphosphate and uridine triphosphate that has been leaked into the extracellular space, nitric oxide, heat shock proteins, S100 proteins, extracellular calcium ion levels, and cytokines (107, 108). The presence of DAMPs on the central side of the blood–brain barrier (BBB) are also associated with endothelial damage, which further contributes to increased permeability and infiltration of immune cells from the periphery (109).

Transcriptomic research has not yet identified pathways involved in the timeline of PSD pathogenesis in bloods; however, an overview of studies examining the whole blood ribonucleic acid (RNA) profiles of different stroke subtypes, including TIA in <1 week, has yielded interesting results (110). Functional analysis of the genes in these studies has shown that immune and homeostasis pathway expression can differentiate between cardioembolic and atherosclerosis stroke (111), and Gene Ontology clustering in cardioembolic ischemic stroke suggests gene expression indicative of cell death, lipid metabolism, and metal ion transport. Thus, here we propose that the proteomically detectable state of the peripheral immune system may be indicative of unresolved ischemic damage in the central parenchyma, resulting in disruptions to inflammatory, metabolic, and homeostatic balance that is related to the transcriptomic profile in the early stages of stroke damage (110). While there is no neurobiological evidence presented in our study, current knowledge of the etiology of PSD suggests that persistent neuroinflammation, driven by an increase in pro-inflammatory but also a decrease in anti-inflammatory cytokine signaling, is responsible for depressive symptoms post-stroke (22, 112). Indeed, it is possible that peripheral immunodepression may be caused by central and upstream molecular cascades that include cross BBB cytokine signaling (113) or bioavailability of immunoglobulin antibodies (114) and leukocyte immune cells (115). Thus, PSD can be considered as a natural sequelae of incomplete recovery from stroke that can be further exacerbated by anxiety from psychosocial issues (8).

We have shown that two innate immune pathways in peripheral bloods, complement and coagulation, trend toward downregulation at the 3-month phase in correlation with mild symptoms of depression. The complement system is largely involved in an array of normal and immunoregulatory functions, although its role in immunodepression post-stroke has not been explored (Table 7). As a whole however, immunodepression after stroke is a well-documented and a natural consequence of ischemia, but is poorly understood in functional terms (116). Initially, it appears that immunodepression is counterproductive after stroke as it may increase the chances of commonly reported secondary infections such as urinary tract infection and pneumonia (117, 118). Additionally, immune suppression is thought to be an adaptive response to central inflammation as an autoimmune response against the brain would be detrimental to recovery outcomes and possibly exacerbate damage (116, 119). There are many factors that may maintain poor immune responses and recovery in animal models such as induced psychosocial stress (120), abnormal BBB permeability (121), and possible ongoing antigen-related responses that have yet to be fully characterized (122, 123).

**Table 7:** **A brief overview of the complement system in both central and peripheral functioning related to stroke risk, the stroke, and post-stroke outcomes, as understood from both human and animal studies**.

	Pre-stroke function and risk factors		Stroke (0–48 h)		Stroke outcomes (48 h+)	
Central	+	C3a regulates neurogenesis *in vitro* by determining differentiation and migration of neural progenitor cells (124)	+	Complement inhibition by various methods such as C5a antagonists (125) and intravenous immunoglobulin (126) are neuroprotective	+	Astrocyte-modulated complement responses enhance the ability of microglia to remove cell death debris (127)
	+	Complement system involved in synaptic pruning by opsonization involving recruitment of microglia (128)	−	Enhances neutrophil adhesion and leukocyte activity which leads to further tissue damage (129)	+	Complement promotes neurogenesis post-cerebral ischemia (130)
			−	Propagation of neuroinflammation and apotopic cell death (131, 132)		
Peripheral	+	Constitutes part of the immune system, involved in clearance of pathogens *via* opsonization and cell lysis. Can initiate a local inflammatory response (50)			−	Early elevation of plasma levels of complement predict negative functional outcome following aneurysmal subarachnoid hemorrhage (133)
	−	Increased serum C4 levels in patients with coronary artery disease predicts stroke risk (134)			−	Early elevation of plasma levels of complement predict negative functional outcome following aneurysmal subarachnoid hemorrhage (133)
	−	C5a induces vasodilation independent of histamine (135) and increased central venous pressure (136)			−	Reduced complement protein expression in blood associated with mild depressive symptoms (current study)
	−	Serum C3 levels independently associated with myocardial infarctions and ischemic events, including TIA (137)				

*The + denoted functions indicate normal or positive functioning, while the − denoted functions indicate counterproductive functioning. Overall, the complement system is involved in regulation of immune function either by localized cell lysis cascades or targeting for removal by phagocytosis. The same is true in both central parenchyma and periphery, where microglia are the primary phagocytes in the brain, while neutrophils, dendritic cells, and monocytes are the primary phagocytes in the periphery. However, this distinction no longer holds in the event of a stroke, where BBB disruption allows some immune cells to infiltrate from the periphery. The complement system exhibits dual roles in the brain, propagating cell death by encouraging the DAMP-initiated signaling cascades of apoptosis but also neuroprotection by removing cellular debris and engaging in synaptic pruning, contributing to post-stroke recovery*.

There has been little research exploring the role of complement and indeed immunity post-stroke in relation to depression. The results here suggest that there is ongoing immunosuppression in the periphery with mild depressive symptoms, even at 3 months post-stroke. Previous research into antigen-presenting cells, such as macrophages and dendritic cells, has established that their expression is elevated centrally, but downregulated peripherally post-stroke (138, 139). A trend toward immune recovery would stipulate that a balance has been reached in these cell levels for both central and periphery. Indeed, it has also been theorized that this discrepancy accounts for the recruitment of peripheral dendritic cells into the brain to maintain a central inflammatory and immune response (140). Thus, given these lines of evidence, it is possible that our current findings reflect an ongoing sub-acute state of pro-inflammation that has not yet transitioned to anti-inflammation (109), manifesting behaviorally as depression symptoms and molecularly as peripheral immunodepression of the complement and coagulation systems.

### Limitations and Future Studies

The patients in this study were not severely depressed and had recovered well after their stroke, with some patients recording a history of pre-stroke depression. Furthermore, there was no age-matched control group present in this study. The current study was a pilot to investigate the feasibility of the proteomics approach in stroke. While we found a significant association even in a sample with mild depressive symptoms and mild stroke severity, future studies that employ larger sample sizes with depressive symptoms and neurological severity that represent the range commonly experienced are recommended. Future studies may consider basic or laboratory assessment of the immunological condition of patients post-stroke as well as the relationship between clinical and/or psychosocial factors on PSD. We used a discovery approach where the findings can stand alone for the stroke cohort, although it is recognized that this approach is not as robust as with age-matched controls. Therefore, we recommend future comparison with age-matched non-stroke controls, with and without depression, to enable a more comprehensive interpretation of findings. In addition, future studies may compare the proteomes of other biofluids, such as CSF and urine, of the same patients to develop a better understanding of the compartmentalization or relationships of BBB and kidney physiology post-stroke. Complement and coagulation functioning can also be assayed in blood in the traditional hematological laboratory setting. From a technical perspective, further studies could optimize the comparison of ionic chelation properties of anticoagulants as this is not well understood in whole blood samples. It is also feasible to employ immunodepletion or different fractionation techniques on the mass spectrometer in the sample preparation stages to resolve dynamic range and peak detection issues for low abundance compounds.

## Conclusion

This study examined the serum proteomic profile of stroke survivors at 3 months post-stroke using a label-free approach. The findings here and in Zhan et al. (47) are complementary and provide a basis for further research into blood proteins recently identified to be involved in the pathophysiology of PSD and possibly in other cerebrovascular diseases with comorbid anxiety. Analysis by GSEA on various databases has revealed enriched gene sets that are identifiable as complement and innate immune processes. As all of these gene sets were negatively enriched when correlated with increasing depressive symptoms of ischemic stroke survivors, this was interpreted as peripheral immunodepression, indicative of unresolved ongoing inflammatory processes in the brain. This interpretation is supported by the substantial body of literature that has linked PSD etiology to overactive immunologic processes, leading to increased inflammatory processes in both peripheral and central compartments. While these findings add to the growing body of evidence for differentially expressed proteins in PSD, more research is needed to characterize their molecular processes and how their expressions may change as result of the stroke and in development of PSD.

## Consent Statement

Patient, family member, or legally responsible person, depending on local ethics requirements, have given informed consent for participation in the START_PrePARE study.

## Author Contributions

Main author: VN. Senior authors: SC and LC. Cohort Project (START and PrePARE) conception and design: LC, GD, SD, DH, SC, LM, and HM. Data analysis application and interpretation: VN, SC, LG, TT, and DH. Proteomics methodology and data acquisition: SC, LC, DH, PF, and IC. Patient testing, project co-ordination: LC, HM and TT. All of the authors listed have read and approved the final version of the manuscript.

## Conflict of Interest Statement

The authors declare that the research was conducted in the absence of any commercial or financial relationships that could be construed as a potential conflict of interest.

## Abbreviations

ApoA4, ApoC2, APOE, apolipoproteins A4, C2, E; BBB, blood–brain barrier; C6, C3, C3a, C5a, complement component 6, 3, 3 a, 5a; EDTA, ethylenediaminetetraacetic acid; ESI, electrospray ionization; FDR, false discovery rate; GO, Gene Ontology; GSEA, gene set enrichment analysis; GSN, gelsolin; ITRAQ, isobaric tagging for relative and absolute quantification; KEGG, Kyoto Encyclopedia of Genes and Genomes; LC, liquid chromatography; LFQ, label-free quantification; MADRS, Montgomery–Åsberg Depression Rating Scale; MAC, membrane attack complex; MS, MS/MS, mass spectrometry, tandem mass spectrometry; MBL2, mannan-binding lectin 2; MoCA, Montreal Cognitive Assessment; mRS, modified Rankin Scale; NES, normalized enrichment score; NIHSS, National Institute of Health Stroke Scale; PHQ-9, PHQ-2, Patient Health Questionnaire 9, 2; PrePARE, Prediction and Prevention to Achieve optimal Recovery Endpoints; PSD, post-stroke depression; RNA, ribonucleic acid; S100A12, S100 calcium-binding protein A12; SIGMA, structured interview guide for the Montgomery–Åsberg Depression Rating Scale; SLE, systemic lupus erythematous; START, STroke imAging, pRevention and Treatment longitudinal stroke cohort study; TF, transferring; TIA, transient ischemic attack; TLR4, toll-like receptor 4; TOAST, trial of ORG 10172 in acute treatment; tPA, tissue plasminogen activator.
